# Shifting Concepts in Esthetic‐Zone Implant Therapy: Reflections From a 15‐Year Institutional Registry

**DOI:** 10.1111/jre.70146

**Published:** 2026-07-06

**Authors:** Miha Pirc, Eugenia Settecase, Margherita Giorgia Liguori, Tobias Janson, Ronald Ernst Jung, Tim Joda

**Affiliations:** ^1^ Clinic of Reconstructive Dentistry, Center for Dental Medicine University of Zurich Zurich Switzerland; ^2^ Department of Reconstructive Dentistry, UZB University Center for Dental Medicine Basel University of Basel Basel Switzerland

**Keywords:** big data, esthetics, guided bone regeneration, health services research, implant dentistry, registry‐based trial

## Abstract

This registry‐based analysis provides a longitudinal overview of implant therapy within the maxillary esthetic zone over a 15‐year period. The data suggest three developments: (1) increasing integration of digital workflows through CAIS; (2) a cautious tendency toward earlier implant placement protocols; (3) a gradual reduction in simultaneous guided bone augmentation.

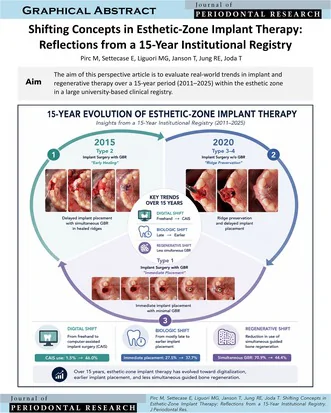

Despite the growing body of evidence derived from randomized controlled trials (RCTs), much of the available data originates from highly controlled clinical environments with strict inclusion criteria. Consequently, the generalizability of these findings to everyday clinical practice may be limited. In addition, the translation of RCT findings into clinical practice often occurs with a considerable time lag [1]. Large‐scale longitudinal registry observations reflecting routine clinical decision‐making, evolving technologies, and changing treatment philosophies over extended periods remain scarce, particularly in academic clinical settings, where innovative concepts are frequently introduced early but applied pragmatically in daily care [2].

In this context, registry‐based research provides a valuable methodological approach to systematically capture and analyze everyday clinical data. Such analyses enable the evaluation of how therapeutic concepts are implemented under real‐world conditions and how treatment strategies evolve over time. By translating large‐scale patient datasets into clinically meaningful insights, registry‐based studies allow the identification of current treatment patterns and may help anticipate future developments in implant therapy [3].

Therefore, the aim of this perspective article is to evaluate real‐world trends in implant and regenerative therapy over a 15‐year period (2011–2025) within the esthetic zone in a university‐based clinical registry, with particular emphasis on implant characteristics, surgical timing strategies, and the use of regenerative procedures.

## 1 Implant Registry

This trend analysis studied routine clinical data derived from the implant registry of the Clinic of Reconstructive Dentistry, Center for Dental Medicine, University of Zurich, Switzerland. The institution represents a highly specialized academic center for implant dentistry. All treatments were performed by experienced faculty members and supervised postgraduate residents. The registry captured clinical data related to implant therapy and regenerative procedures, thereby reflecting routine clinical decision‐making. The primary purpose of the registry was to document treatment characteristics, surgical workflows, implant‐related variables, and regenerative procedures for clinical quality assurance and longitudinal evaluation of evolving treatment concepts, rather than to serve as a comprehensive outcomes registry. The implant registry was established in 2002 as an institutional clinical quality assurance and research database. Data were entered prospectively as part of routine clinical documentation using predefined variables and standardized recording procedures, underwent regular plausibility and completeness checks, and were subsequently migrated to a structured REDCap environment in 2024 to further enhance consistency of longitudinal data management. The present analysis comprised all patients requiring implants in the maxillary esthetic region (15–25) over a 15‐year observation period (2011–2025). Patients were included consecutively, independent of implant system or surgical protocol. The registry is compliant with Swiss data protection regulations, the Federal Human Research Act, and international recommendations for health registries, including FAIR principles for data stewardship. The implant registry has been maintained since 2002 as part of institutional clinical documentation and quality assurance procedures. Ethical approval for the retrospective scientific analysis and publication of anonymized registry data was obtained from the Cantonal Ethics Committee of Zurich (AO_2025–00094).

The following variables were included for the present analysis:

- Implant site and position;
- Implant characteristics, grouped as length (short < 8.0 mm, regular ≥ 8.0 mm) and diameter (reduced diameter < 4.0 mm, regular ≥ 4.0 mm);
- Surgical protocol, as freehand (FH) and computer‐assisted implant surgery (CAIS):
- Timing of implant placement (Types 1–4) [4];
- Simultaneous bone regeneration procedures, as a type of technique, materials, and defect morphology (contour defects, fenestrations, intra‐osseous or combined defects).

All analyses were performed using R statistical software (version 4.5.2; R Core Team, 2025). Missing values were reported descriptively and no imputation procedures were performed, consistent with the exploratory nature of the present perspective analysis. Descriptive statistics were used to summarize variables related to implants, sites, and surgical protocols across the study period (2011–2025). To explore temporal trends in implant and treatment‐related characteristics, annual proportions were calculated and visually inspected using exploratory plots. These annual proportions were additionally summarized using median annual percentages and interquartile ranges (IQRs) across the observation period. In addition to visual inspection of temporal trends, exploratory Cochran–Armitage trend tests were conducted to assess linear trends across three consecutive 5‐year periods (2011–2015, 2016–2020, and 2021–2025) for cases involving CAIS, immediate (Type 1) implant placement, and simultaneous GBR. Smoothed LOESS trend plots with 95% bootstrap confidence intervals are provided in the Supporting Information (Figures S1–S3).

## 2 Trend Analysis

To facilitate interpretation of temporal developments and site‐specific differences, findings focused on two complementary analytical frameworks. First, overall trends in implant therapy were evaluated across three consecutive 5‐year periods, allowing the identification of gradual shifts in treatment strategies over time. Second, analyses were stratified according to implant position, comparing incisor (13–23) and premolar (15,14,24,25) sites within the maxillary esthetic zone. Tables 1 and 2 provide comprehensive overviews of the patient and implant characteristics as well as surgical protocols including regenerative procedures.

**TABLE 1:** Demographics, implant characteristics, surgical protocol including implant timing.

Patient (*N* = 2575) and implant (*N* = 2945) characteristics	Category	*N* (%)
Gender	Female	1166 (45.3)
	Male	1126 (43.7)
	Missing data	283 (11.0)
Age cohorts	20–40 years	63 (2.4)
	41–60 years	432 (16.8)
	61–80 years	884 (34.3)
	> 80 years	982 (38.1)
	Missing data	214 (8.3)
Implant site	Incisors (13–23)	1513 (51.4)
	Premolars (15,14,24,25)	1432 (48.6)
Implant length	Short (< 8.0 mm)	142 (4.8)
	Regular (≥ 8.0 mm)	2764 (93.9)
	Missing data	39 (1.3)
Implant diameter	Narrow (< 4.0 mm)	1018 (34.6)
	Regular (≥ 4.0 mm)	1897 (64.4)
	Missing data	30 (1.0)
Surgical protocol	Freehand (FH)	1361 (46.2)
	Computer‐assisted implant surgery (CAIS)	1584 (53.8)
Implant timing	Type 1	219 (7.4)
	Type 2	282 (9.6)
	Type 3	334 (11.3)
	Type 4	2059 (69.9)
	Missing data	51 (1.7)

**TABLE 2:** Implants requiring simultaneous bone augmentation and regenerative materials used.

Implants (*N* = 2945)	Category	*N* (%)
Bone augmentation procedures (simultaneous GBR)	No	1279 (43.4)
	Yes	1666 (56.6)
	*Bone defect morphology*	
	Contour (=dehiscence)	900 (54.0)
	Fenestration	185 (11.1)
	Sinus floor elevation (SFE)	148 (8.9)
	Intra‐Osseous	139 (8.3)
	Combined	132 (7.9)
	Unknown	162 (9.7)
	*Bone augmentation materials*	
	Autogenous	20 (1.2)
	Autogenous + DBBM Granules	45 (2.7)
	DBBM Granules	583 (35.0)
	DBBM Granules + DBBMC	251 (15.1)
	DBBMC	331 (19.9)
	DBBM Block	51 (3.1)
	Other	10 (0.6)
	Missing	375 (22.5)
	*Membranes*	
	Non‐resorbable	29 (1.7)
	Resorbable	1305 (78.3)
	Missing data	332 (19.9)

Abbreviations: DBBM, deproteinized bovine bone mineral; DBBMC, deproteinized bovine bone mineral plus collagen.

Analyzed implants were evenly distributed between incisor (50.6%) and premolar sites (49.4%) (Figure 1). Considering implant characteristics, regular‐length implants (≥ 8 mm) and implants with a regular diameter (≥ 4.0 mm) were mostly placed (95.1% and 65.1%) (Table 3). In an exploratory mixed‐effects logistic regression, accounting for clustering of multiple implants within patients, implant diameter and length were significantly associated with implant site. Regular diameter implants showed higher odds in premolar rather than incisor sites (OR: 2.24, CI95 1.85–2.70; *p* < 0.001). In contrast, implants with a regular length exhibited lower odds of placement in premolar sites compared to incisors (OR: 0.16, CI95 0.10–0.27; *p* < 0.001), indicating that shorter implants were used relatively more often for premolar replacements.

**FIGURE 1:**
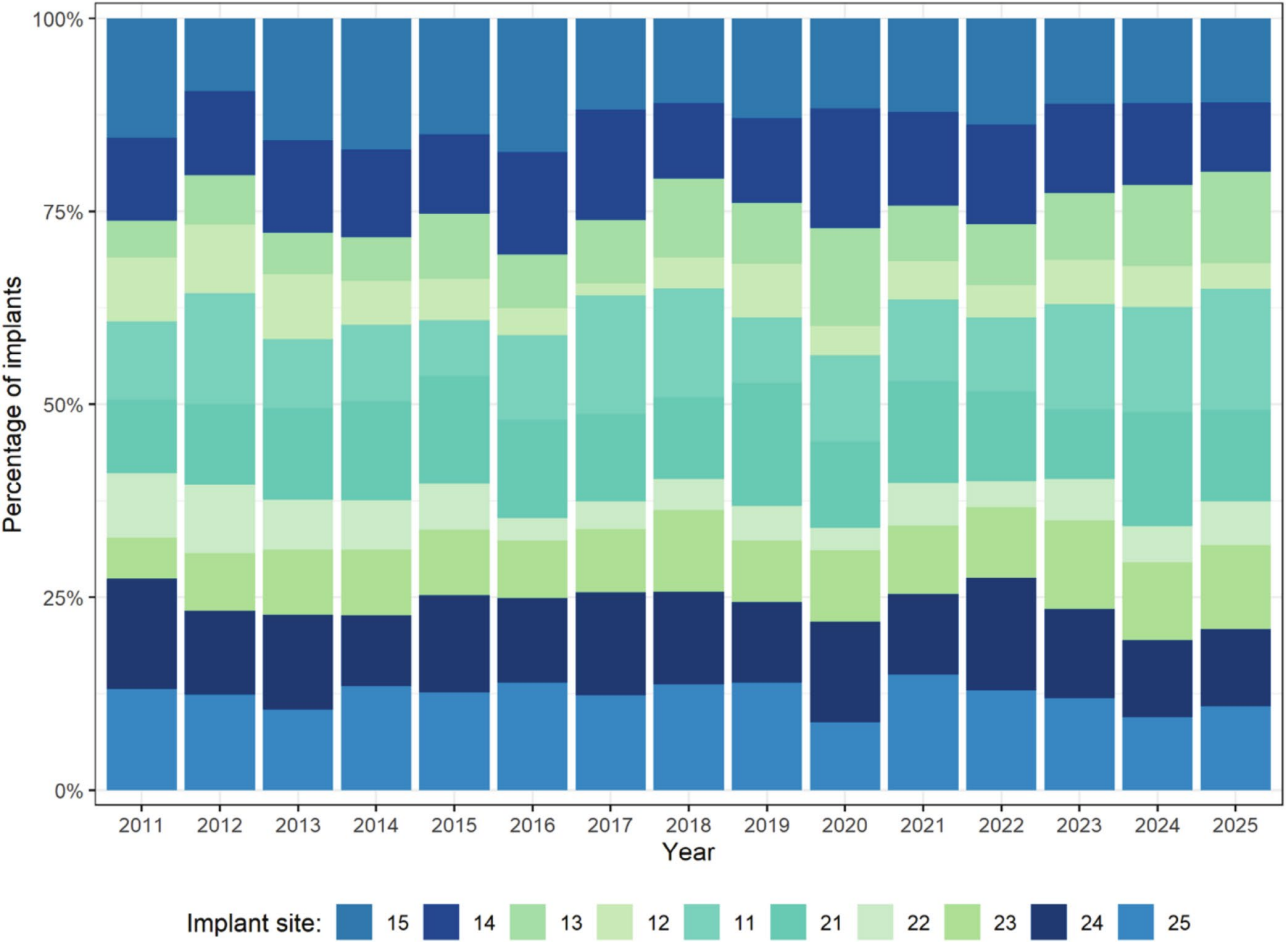
Distribution of implants placed in the maxillary esthetic region by specific site over time.

**TABLE 3:** Percentage of implants placed in incisor (13–23) and premolar (15, 14, 24, 25) sites, categorized by length and diameter.

Implant	2011–2015		2016–2020		2021–2025		Total	
Characteristics	Incisors (%)	Premolars (%)	Incisors (%)	Premolars (%)	Incisors (%)	Premolars (%)	Incisors (%)	Premolars (%)
Length								
Short (< 8.0 mm)	1.4	8.1	1.0	7.9	2.5	8.9	1.7	8.3
Regular (≥ 8.0 mm)	98.6	91.9	99.0	92.1	97.5	91.1	98.3	91.7
Diameter								
Narrow (< 4.0 mm)	38.9	17.2	44.8	30.7	42.9	28.7	42.7	26.7
Regular (≥ 4.0 mm)	61.1	82.8	55.2	69.3	57.1	71.3	57.3	73.3

A shift in surgical protocol was observed during the overall period. In early years of analysis (2011–2015), FH was the main surgical approach (77.0%). From 2016 onward, a continuous increase of CAIS was observed, and in the most recent years (2021–2025), CAIS became the principal surgical approach (75.7%) (Figure 2). The increase in CAIS adoption across the observation period was statistically significant (Cochran–Armitage *Z* = 21.84, *p* < 0.001; Figure S1).

**FIGURE 2:**
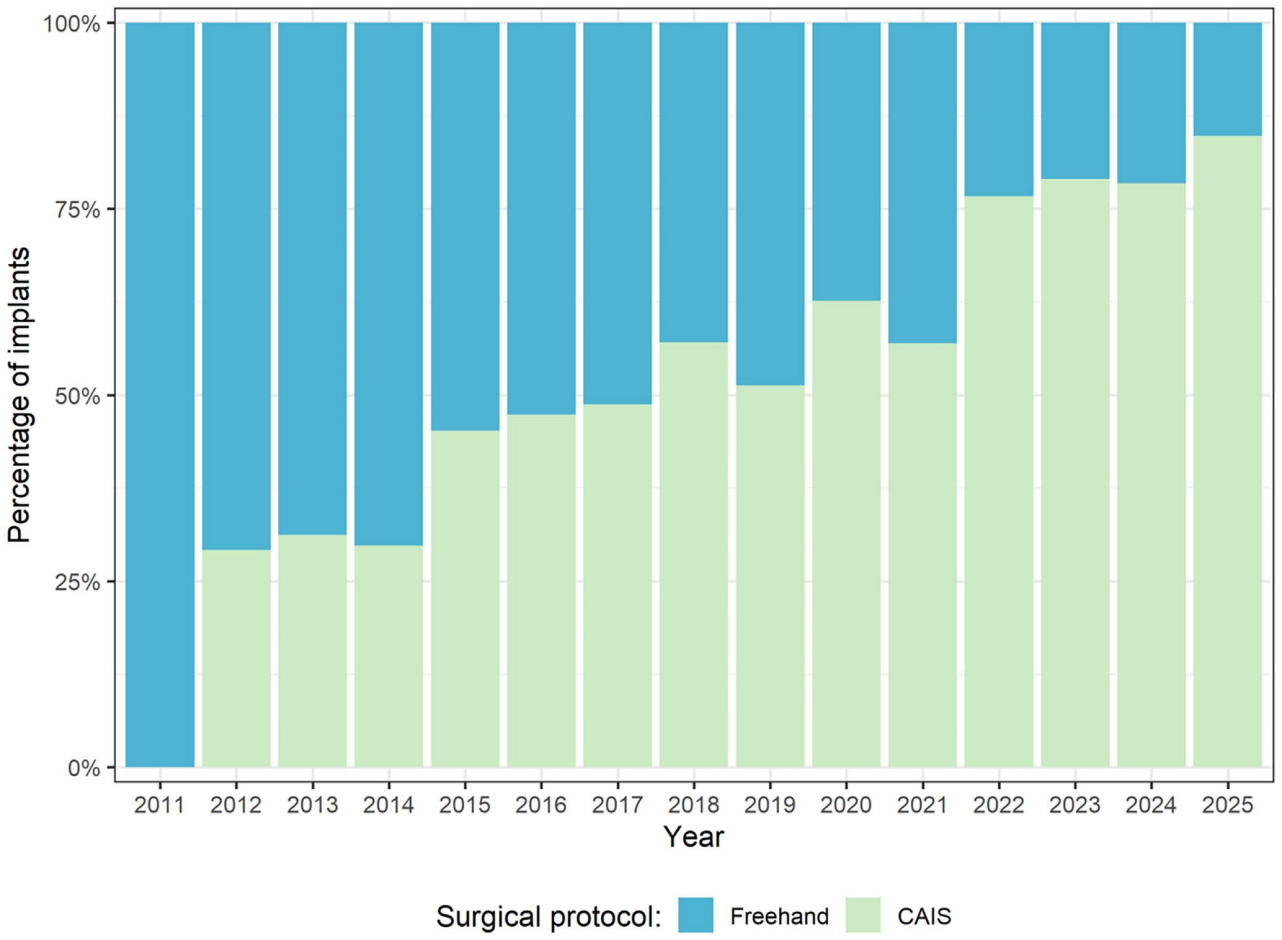
Annual percentage of implants placed using freehand (FH) or computer‐assisted implant surgery (CAIS).

Late implant placement (Type 4) represented the most frequently applied protocol throughout the entire observation period (71.2%). Immediate (Type 1), early (Type 2), and early‐delayed (Type 3) implant placement protocols collectively accounted for the remaining percentage (28.8%). Type 3 protocol was more common in the early years, whereas the Type 1 protocol became more prevalent in the later years. Type 2 remained relatively stable overall, though all three protocols exhibited annual fluctuations (Figure 3). Stratification by location revealed that incisor sites were more commonly associated with immediate and early placement protocols, meanwhile premolar sites were more often related to late placement. Although the association between implant position and timing was statistically significant (*p* < 0.001), its strength was small (Cramer's *V* = 0.160). Exploratory trend analysis showed a significant increase in immediate implant placement over time (Cochran–Armitage *Z* = 3.92, *p* < 0.001). However, this increase was much smaller than the trends observed for CAIS (*Z* = 21.84) and GBR (*Z* = −11.39). Immediate (Type 1) implant placement remained relatively low throughout the studied period, increasing from 4.2% in 2011–2015 to 9.3% in 2021–2025. These findings suggest a gradual and cautious shift toward earlier implant placement (Figure S2).

**FIGURE 3:**
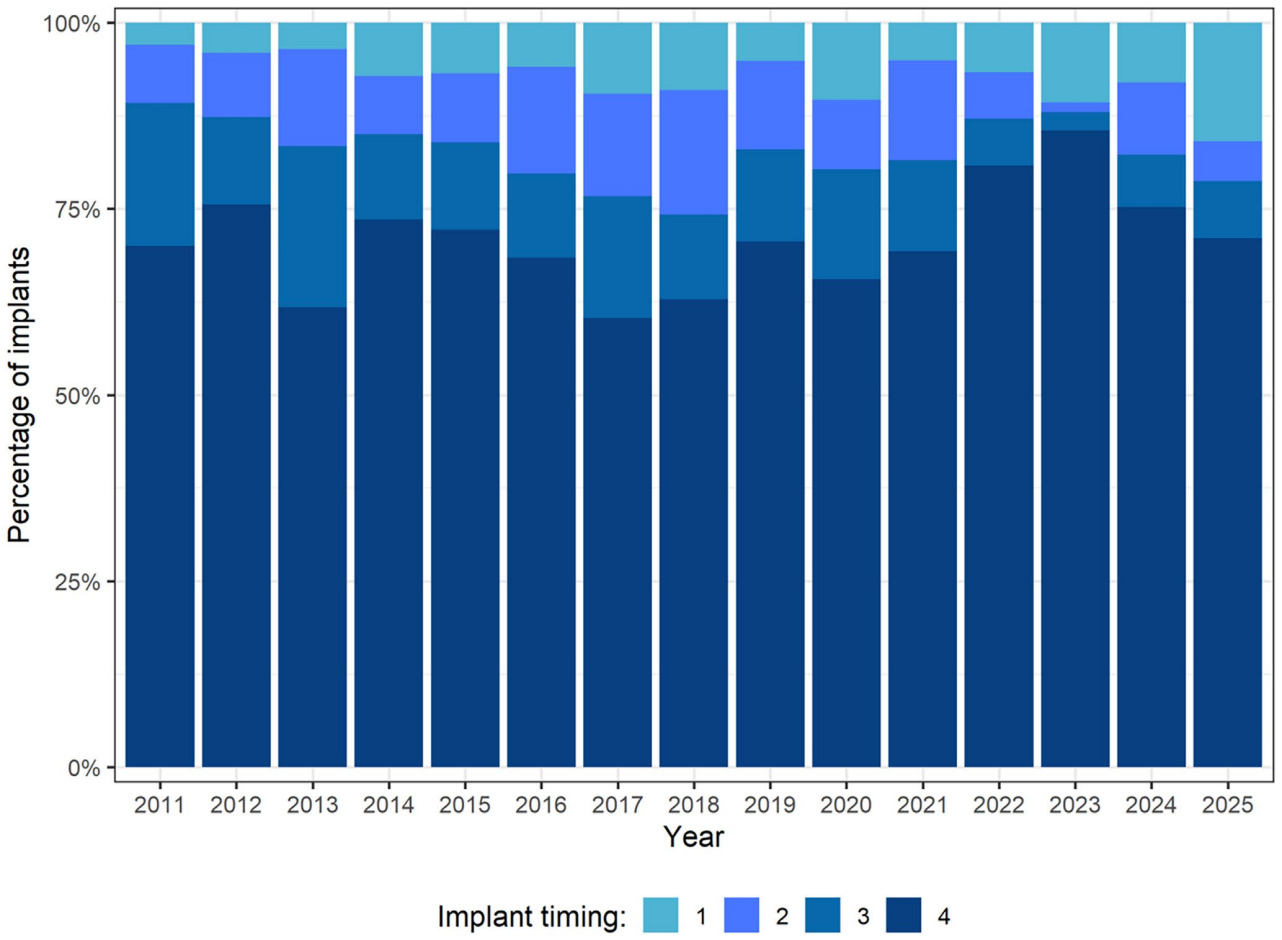
Distribution of surgical implant timing protocols (Types 1–4).

Across the entire study period, a substantial proportion of implants were placed in combination with bone augmentation procedures. In the earlier years (2011–2015), simultaneous guided bone regeneration (GBR) was performed in 70.9% of implant placements. Over time, a gradual reduction in the relative frequency of GBR was observed, declining to 44.4% in recent years (2021–2025) (Figure 4). The decline was statistically significant (Cochran–Armitage *Z* = −11.39, *p* < 0.001; logistic regression OR per year = 0.90, 95% CI 0.88–0.92, *p* < 0.001; Figure S3). The temporal trends in regenerative procedures likely reflect an evolution in indication rather than opposing developments. Earlier periods were characterized by Type 2 implant placement frequently combined with conventional GBR for defect reconstruction. With increasing implementation of alveolar ridge preservation, the need for extensive bone augmentation decreased. More recently, immediate implant protocols have been accompanied by renewed concomitant grafting, primarily to manage peri‐implant jumping gaps and support contour preservation rather than reconstruct advanced defects. Incisor sites consistently demonstrated a higher percentage of simultaneous GBR compared with the premolar region. This site‐specific distribution remained stable throughout the observation period. Among cases requiring GBR, the percentage of deproteinized bovine bone mineral (DBBM) decreased continuously, from 45.3% (2011–2015) to 15.8% (2021–2025); meanwhile, an increase in the use of deproteinized bovine bone mineral plus collagen (DBBMC) was observed, from 17.5% (2011–2015) to 49.5% (2021–2025) (Figure 5).

**FIGURE 4:**
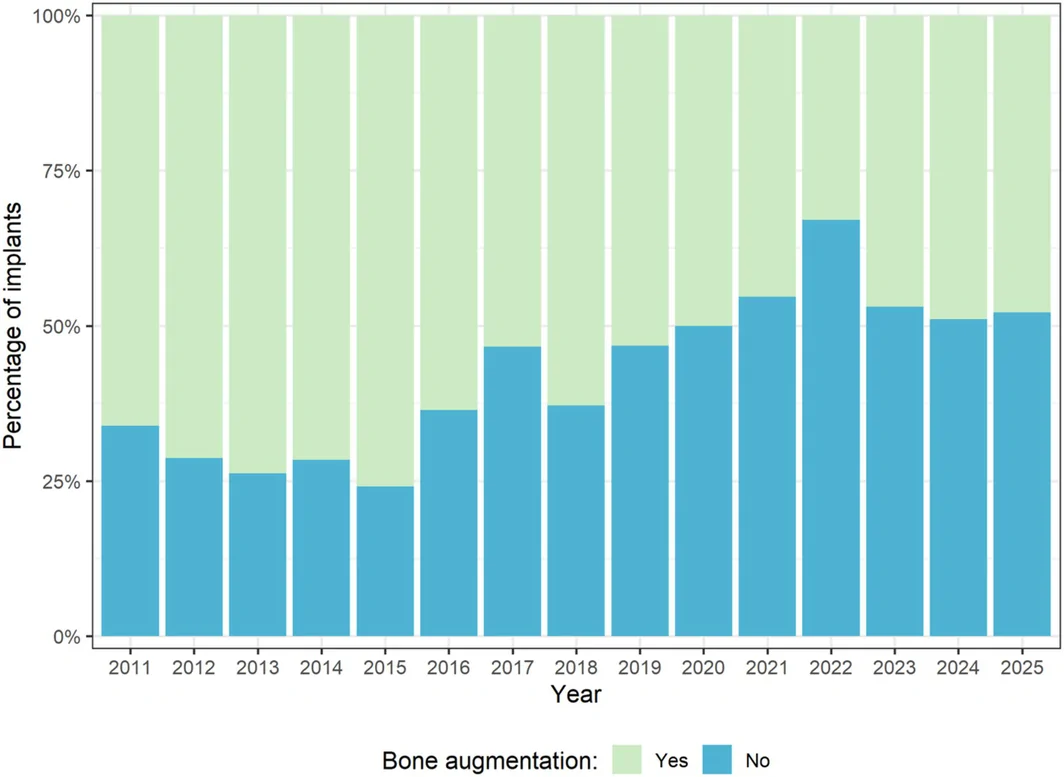
Annual percentage of implants placed with and without simultaneous bone augmentation (GBR).

**FIGURE 5:**
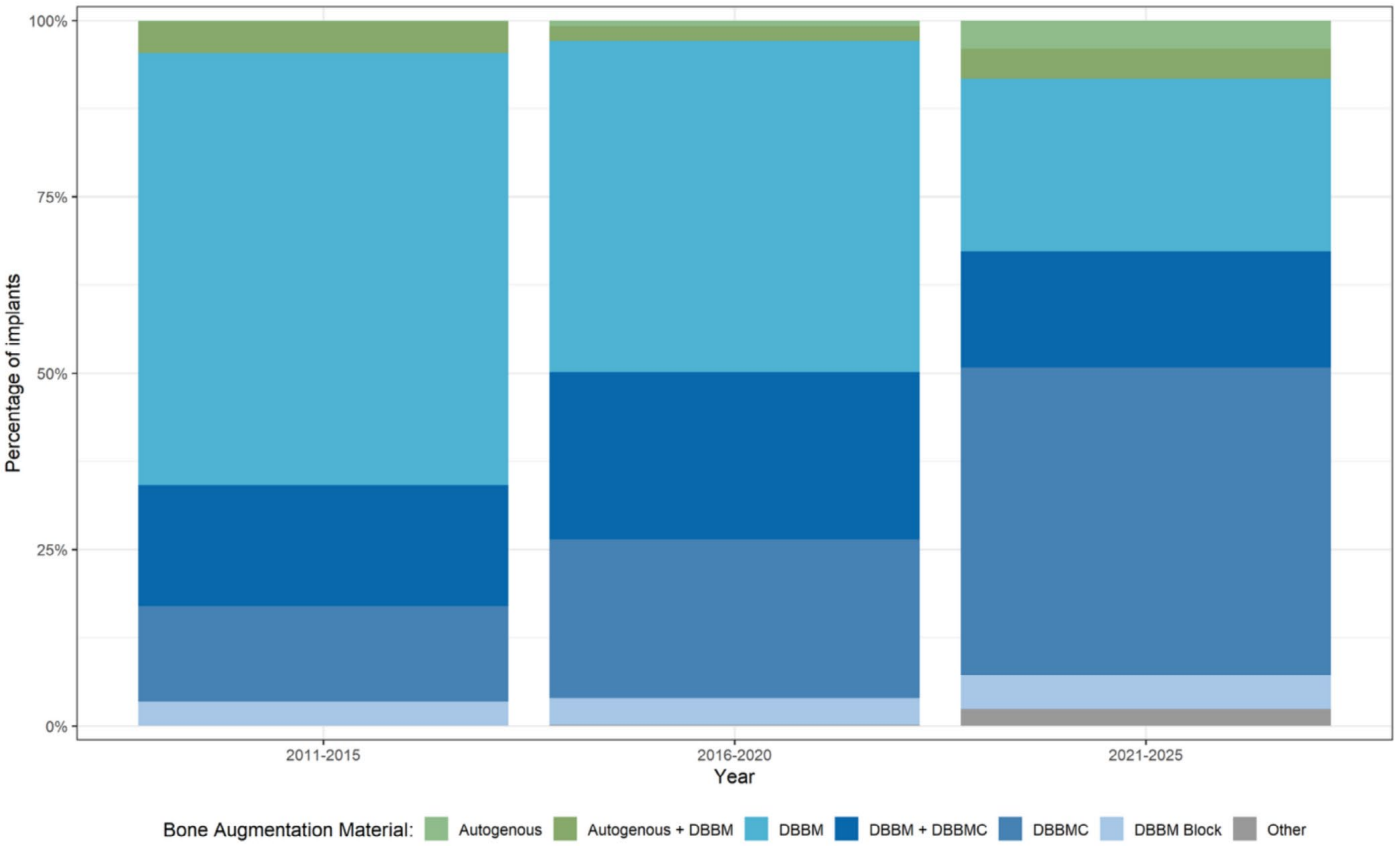
Overview of the different bone augmentation materials grouped by the time blocks from 2011 to 2015, 2016 to 2020, and 2021 to 2025. DBBM, deproteinized bovine bone mineral; DBBMC, deproteinized bovine bone mineral plus collagen.

The frequency and morphology of bone defects varied according to the timing of implant surgery. Simultaneous GBR was mostly associated with immediate (Type 1) and early (Type 2) protocols, whereas implants placed in healed sites (Type 4) revealed a comparatively lower need for regenerative procedures. Among cases requiring GBR, contour defects (=dehiscence) represented the most common defect configuration (54.0%), while fenestration, intra‐osseous, and combined defects were observed less frequently. A statistically significant moderate association between defect morphology and timing was identified (*p* < 0.001, Cramer's *V* = 0.269).

## 3 Conclusion

The present registry‐based analysis provides a longitudinal overview of implant therapy within the maxillary esthetic zone over a 15‐year period in a specialized monocentric university setting. Beyond descriptive trends, the data suggest three concurrent developments in contemporary implant therapy (Figure 6):

1. increasing integration of digital workflows through computer‐assisted implant surgery (CAIS);
2. a cautious tendency toward earlier implant placement protocols; and
3. a gradual reduction in simultaneous guided bone augmentation.

**FIGURE 6:**
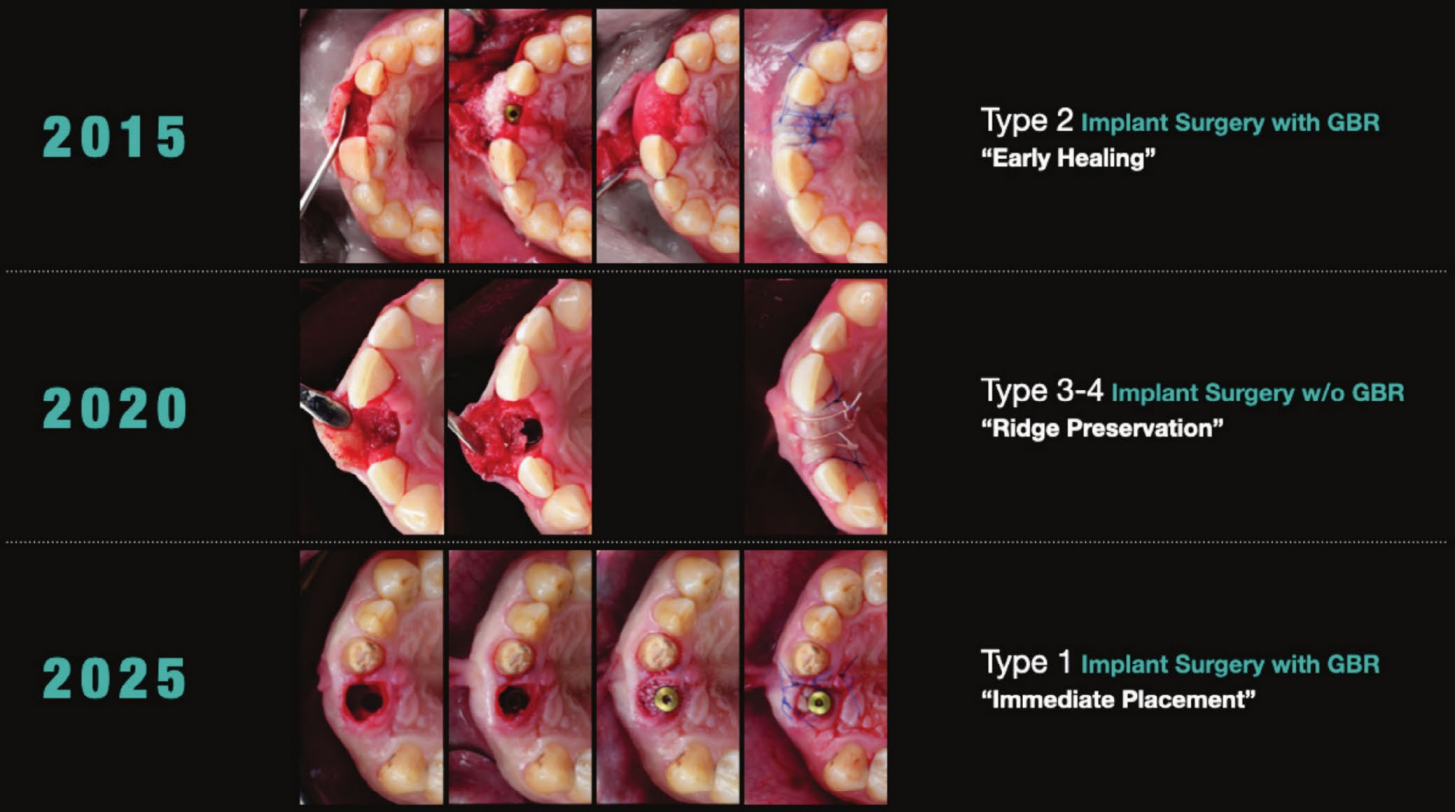
Summary of prevailing treatment patterns across the three consecutive observation periods and illustration of a gradual yet consistent shift in clinical decision‐making with the introduction and integration of new therapeutic concepts. During the first period (2011–2015), the predominant treatment approach consisted of early implant placement (Type 2) associated with simultaneous GBR (upper panel). In the second period (2016–2020), the increasing implementation of alveolar ridge preservation procedures was associated with a shift toward later implant placement protocols (Types 3 and 4), mostly performed without simultaneous GBR (middle panel). In the most recent period (2021–2025), a further transition toward immediate implant placement (Type 1), frequently combined again with concomitant GBR, can be observed (lower panel).

Together, these developments illustrate how technological innovation, biological understanding, and clinical pragmatism collectively shape therapeutic strategies in daily practice [5]. Whether the observed developments represent true paradigm shifts or rather gradual refinements of established concepts remains open to debate. The present observations, however, suggest that implant therapy evolves predominantly through adaptive integration of technological innovation, biomaterial development, and increasing biological understanding rather than through abrupt replacement of previous protocols. In this context, specialized academic centers may function as early adopters, where novel concepts are critically evaluated and selectively implemented before broader translation into routine clinical practice.

The major strength of this trend analysis lies in the comprehensive inclusion of consecutively treated patients within a defined anatomical region over a long observation period. The registry‐based design minimizes selection bias and provides valuable insight into routine clinical decision‐making. However, the present observations originate from a single highly specialized academic referral center and may therefore reflect institutional philosophies and early adoption of innovations rather than routine implant dentistry in general practice. In addition, the registry was primarily designed to document treatment characteristics, surgical workflows, and regenerative procedures rather than comprehensive longitudinal outcome measures. Although adverse events can be recorded, systematic assessment of long‐term biological, technical, and patient‐reported outcomes was beyond the scope of the present registry infrastructure. Furthermore, long‐term clinical outcomes, patient‐reported measures, and potentially relevant confounders, such as soft‐tissue phenotype, smoking status, systemic conditions, or prosthetic variables, were not assessed. Consequently, the findings should be interpreted as longitudinal observations of evolving treatment concepts within a specialized environment rather than broadly generalizable trends in implant therapy. The exploratory trend tests were uni‐variable and did not adjust for potential confounders. Given the large sample size (*N* = 2945 implants), even modest temporal changes may reach statistical significance; accordingly, emphasis should be placed on the direction and magnitude of the observed effects rather than on *p*‐values alone.

Importantly, the value of clinical registries extends beyond the description of treatment frequencies and temporal trends. Depending on their structure and predefined purpose, registries can provide insight into how innovations are adopted, how treatment concepts evolve, and how evidence is translated into clinical practice under routine conditions. At the same time, registries are inherently limited by the variables they capture and cannot substitute for controlled clinical trials when causal relationships or treatment efficacy need to be established. Rather, they should be viewed as complementary evidence‐generating infrastructures that support hypothesis generation, quality assurance, implementation research, and the identification of clinically relevant questions for future prospective investigations [3].

While classical clinical trial designs such as randomized controlled trials (RCTs) remain essential to establish efficacy under controlled conditions, they may only partially reflect the complexity of routine clinical decision‐making [3]. In this context, registry‐based infrastructures provide complementary longitudinal evidence by capturing how treatment concepts are implemented, adapted, and translated into clinical practice over time [6]. Future developments may extend beyond descriptive trend analyses toward integration of longitudinal clinical outcomes, patient‐reported measures, AI‐assisted analytics, predictive modeling, and personalized treatment recommendations. Furthermore, harmonized multicenter registries may facilitate collaborative research frameworks, improve external validity, and support pragmatic evidence generation under real‐world conditions [2]. Ultimately, the integration of real‐world data into clinical research frameworks may support more informed, data‐driven decision‐making and foster the development of collaborative, international research networks in implant dentistry.

## Author Contributions

**Miha Pirc:** conceptualization, methodology, investigation, writing – original draft, writing – review and editing, visualization. **Eugenia Settecase:** conceptualization, data curation, software, validation, writing – original draft, writing – review and editing, visualization. **Margherita Giorgia Ligouri:** writing – review and editing, visualization. **Tobias Janson:** data curation, writing – review and editing. **Ronald Ernst Jung:** writing – review and editing. **Tim Joda:** conceptualization, methodology, investigation, supervision, writing – original draft, writing – review and editing, visualization.

## Disclosure

The authors have nothing to report.

## Conflicts of Interest

The authors declare no conflicts of interest.

## Supporting information


**Figure S1:** Annual percentage of implants placed using computer‐assisted implant surgery (CAIS) with smooth LOESS curve.
**Figure S2:** Annual percentage of implants placed In an early timing protocol (Type 1) with smooth LOES curve.
**Figure S3:** Annual percentage of implants placed with simultaneous bone augmentation (GBR), with smooth LOES curve.

## Data Availability

The data that supports the findings of this study are available in the Supporting Information of this article.
