# Supplementary material for: Shifting Concepts in Esthetic‐Zone Implant Therapy: Reflections From a 15‐Year Institutional Registry
Source: J Periodontal Res. 2026 Jul 6;61(9):1009–16. doi: 10.1111/jre.70146 (PMC13768986; doi:10.1111/jre.70146)
Supplement: Supplementary file 1 [file JRE-61-1009-s001.docx]

**SUPLEMENTARY FIGURE S1**

*Figure S1 –Annual percentage of implants placed using computer-assisted implant surgery (CAIS) with smooth LOESS curve.*

**SUPLEMENTARY FIGURE S2**

*Figure S2 –* *Annual percentage of implants placed In an early timing protocol (Type 1) with smooth LOES curve.*

**SUPLEMENTARY FIGURE S3**

*Figure S3 –* *Annual percentage of implants placed with simultaneous bone augmentation (GBR), with smooth LOES curve.*
